# Supplementary material for: Cumulative advantage and citation performance of repeat authors in scholarly journals
Source: PLoS One. 2022 Apr 13;17(4):e0265831. doi: 10.1371/journal.pone.0265831 (PMC9007338; doi:10.1371/journal.pone.0265831)
Supplement: S1 Table — (DOCX) [file pone.0265831.s001.docx]

| \| **Publication order** \| **Count** \| **Percentage** \| **Count** \| **Percentage** \| **Count** \| **Percentage** \| **Count** \| **Percentage** \| **Count** \| **Percentage** \| \| --- \| --- \| --- \| --- \| --- \| --- \| --- \| --- \| --- \| --- \| --- \| \| **0-50%** \| \| **50-75%** \| \| **75-90%** \| \| **90-100%** \| \| **Elite** \| \| \| 1 \| 27476 \| 67.69% \| 22230 \| 63.83% \| 18002 \| 64.21% \| 12834 \| 60.57% \| 6226 \| 55.38% \| \| 2 \| 5801 \| 14.29% \| 5271 \| 15.13% \| 4342 \| 15.49% \| 3567 \| 16.83% \| 1939 \| 17.25% \| \| 3 \| 2559 \| 6.30% \| 2504 \| 7.19% \| 2063 \| 7.36% \| 1735 \| 8.19% \| 1025 \| 9.12% \| \| 4 \| 1383 \| 3.41% \| 1475 \| 4.24% \| 1136 \| 4.05% \| 1009 \| 4.76% \| 598 \| 5.32% \| \| 5 \| 891 \| 2.20% \| 951 \| 2.73% \| 724 \| 2.58% \| 621 \| 2.93% \| 410 \| 3.65% \| \| 6 \| 622 \| 1.53% \| 641 \| 1.84% \| 485 \| 1.73% \| 398 \| 1.88% \| 286 \| 2.54% \| \| 7 \| 459 \| 1.13% \| 458 \| 1.32% \| 333 \| 1.19% \| 278 \| 1.31% \| 204 \| 1.81% \| \| 8 \| 328 \| 0.81% \| 328 \| 0.94% \| 241 \| 0.86% \| 205 \| 0.97% \| 152 \| 1.35% \| \| 9 \| 261 \| 0.64% \| 265 \| 0.76% \| 182 \| 0.65% \| 150 \| 0.71% \| 115 \| 1.02% \| \| 10 \| 215 \| 0.53% \| 200 \| 0.57% \| 145 \| 0.52% \| 115 \| 0.54% \| 85 \| 0.76% \| \| 11 \| 176 \| 0.43% \| 152 \| 0.44% \| 116 \| 0.41% \| 93 \| 0.44% \| 66 \| 0.59% \| \| 12 \| 144 \| 0.35% \| 119 \| 0.34% \| 90 \| 0.32% \| 69 \| 0.33% \| 49 \| 0.44% \| \| 13 \| 112 \| 0.28% \| 93 \| 0.27% \| 72 \| 0.26% \| 50 \| 0.24% \| 35 \| 0.31% \| \| 14 \| 88 \| 0.22% \| 77 \| 0.22% \| 59 \| 0.21% \| 38 \| 0.18% \| 29 \| 0.26% \| \| 15 \| 73 \| 0.18% \| 63 \| 0.18% \| 46 \| 0.16% \| 27 \| 0.13% \| 24 \| 0.21% \| |
| --- | --- | --- | --- | --- | --- | --- | --- | --- | --- | --- | --- | --- | --- | --- | --- | --- | --- | --- | --- | --- | --- | --- | --- | --- | --- | --- | --- | --- | --- | --- | --- | --- | --- | --- | --- | --- | --- | --- | --- | --- | --- | --- | --- | --- | --- | --- | --- | --- | --- | --- | --- | --- | --- | --- | --- | --- | --- | --- | --- | --- | --- | --- | --- | --- | --- | --- | --- | --- | --- | --- | --- | --- | --- | --- | --- | --- | --- | --- | --- | --- | --- | --- | --- | --- | --- | --- | --- | --- | --- | --- | --- | --- | --- | --- | --- | --- | --- | --- | --- | --- | --- | --- | --- | --- | --- | --- | --- | --- | --- | --- | --- | --- | --- | --- | --- | --- | --- | --- | --- | --- | --- | --- | --- | --- | --- | --- | --- | --- | --- | --- | --- | --- | --- | --- | --- | --- | --- | --- | --- | --- | --- | --- | --- | --- | --- | --- | --- | --- | --- | --- | --- | --- | --- | --- | --- | --- | --- | --- | --- | --- | --- | --- | --- | --- | --- | --- | --- | --- | --- | --- | --- | --- | --- | --- | --- | --- | --- | --- | --- | --- | --- | --- | --- | --- | --- | --- |

Table S1. Cumulative Distributions of Repeat Authors for Economics Journals.
