# Supplementary material for: Cumulative advantage and citation performance of repeat authors in scholarly journals
Source: PLoS One. 2022 Apr 13;17(4):e0265831. doi: 10.1371/journal.pone.0265831 (PMC9007338; doi:10.1371/journal.pone.0265831)
Supplement: S2 Table — (DOCX) [file pone.0265831.s002.docx]

| **Publication order** | **Count** | **Percentage** | **Count** | **Percentage** | **Count** | **Percentage** |
| --- | --- | --- | --- | --- | --- | --- |
|  | ***NATURE*** | | ***PNAS*** | | ***SCIENCE*** | |
| 1 | 9340 | 47.77% | 21541 | 36.44% | 12177 | 51.96% |
| 2 | 3276 | 16.75% | 9219 | 15.60% | 3834 | 16.36% |
| 3 | 1900 | 9.72% | 5948 | 10.06% | 2121 | 9.05% |
| 4 | 1265 | 6.47% | 4321 | 7.31% | 1354 | 5.78% |
| 5 | 899 | 4.60% | 3295 | 5.57% | 954 | 4.07% |
| 6 | 674 | 3.45% | 2658 | 4.50% | 699 | 2.98% |
| 7 | 510 | 2.61% | 2201 | 3.72% | 515 | 2.20% |
| 8 | 396 | 2.03% | 1875 | 3.17% | 414 | 1.77% |
| 9 | 311 | 1.59% | 1641 | 2.78% | 334 | 1.43% |
| 10 | 250 | 1.28% | 1440 | 2.44% | 263 | 1.12% |
| 11 | 204 | 1.04% | 1249 | 2.11% | 219 | 0.93% |
| 12 | 165 | 0.84% | 1089 | 1.84% | 180 | 0.77% |
| 13 | 139 | 0.71% | 959 | 1.62% | 147 | 0.63% |
| 14 | 117 | 0.60% | 872 | 1.48% | 123 | 0.52% |
| 15 | 107 | 0.55% | 804 | 1.36% | 100 | 0.43% |

Table S2. Cumulative Distributions of Repeat Authors for *Nature*/*Science*/*PNAS*.
