# Supplementary material for: Cumulative advantage and citation performance of repeat authors in scholarly journals
Source: PLoS One. 2022 Apr 13;17(4):e0265831. doi: 10.1371/journal.pone.0265831 (PMC9007338; doi:10.1371/journal.pone.0265831)
Supplement: S3 Table — (DOCX) [file pone.0265831.s003.docx]

| **Pub. order** | **Intercept** | **Coefficient** | **Std. Error** | **Intercept** | **Coefficient** | **Std. Error** | **Intercept** | **Coefficient** | **Std. Error** | **Intercept** | **Coefficient** | **Std. Error** | **Intercept** | **Coefficient** | **Std. Error** |
| --- | --- | --- | --- | --- | --- | --- | --- | --- | --- | --- | --- | --- | --- | --- | --- |
|  | **0-50%** | | | **50-75%** | | | **75-90%** | | | **90-100%** | | | **Elite** | | |
| 2 | 0.221 | -0.169 | 0.014 | 0.173 | -0.112 | 0.014 | 0.156 | -0.136 | 0.016 | 0.079 | -0.100 | 0.017 | 0.119 | -0.157 | 0.023 |
| 3 | 0.221 | -0.152 | 0.013 | 0.173 | -0.102 | 0.014 | 0.156 | -0.127 | 0.015 | 0.079 | -0.106 | 0.017 | 0.119 | -0.178 | 0.021 |
| 4 | 0.221 | -0.130 | 0.020 | 0.173 | -0.147 | 0.020 | 0.156 | -0.140 | 0.022 | 0.079 | -0.138 | 0.024 | 0.119 | -0.232 | 0.029 |
| 5 | 0.221 | -0.146 | 0.019 | 0.173 | -0.111 | 0.019 | 0.156 | -0.164 | 0.021 | 0.079 | -0.136 | 0.023 | 0.119 | -0.248 | 0.029 |
| 6 | 0.221 | -0.133 | 0.021 | 0.173 | -0.174 | 0.022 | 0.156 | -0.185 | 0.024 | 0.079 | -0.109 | 0.027 | 0.119 | -0.185 | 0.033 |
| 7 | 0.221 | -0.134 | 0.023 | 0.173 | -0.071 | 0.023 | 0.156 | -0.151 | 0.026 | 0.079 | -0.098 | 0.030 | 0.119 | -0.348 | 0.035 |
| 8 | 0.221 | -0.246 | 0.022 | 0.173 | -0.167 | 0.022 | 0.156 | -0.209 | 0.025 | 0.079 | -0.155 | 0.028 | 0.119 | -0.381 | 0.035 |
| 9 | 0.221 | -0.202 | 0.035 | 0.173 | -0.225 | 0.036 | 0.156 | -0.129 | 0.039 | 0.079 | -0.187 | 0.042 | 0.119 | -0.270 | 0.049 |
| 10 | 0.221 | -0.189 | 0.026 | 0.173 | -0.192 | 0.026 | 0.156 | -0.242 | 0.029 | 0.079 | -0.163 | 0.032 | 0.119 | -0.306 | 0.039 |
| 11 | 0.221 | -0.178 | 0.037 | 0.173 | -0.213 | 0.038 | 0.156 | -0.213 | 0.042 | 0.079 | -0.142 | 0.047 | 0.119 | -0.485 | 0.053 |
| 12 | 0.221 | -0.249 | 0.042 | 0.173 | -0.153 | 0.044 | 0.156 | -0.237 | 0.049 | 0.079 | -0.104 | 0.056 | 0.119 | -0.573 | 0.063 |
| 13 | 0.221 | -0.371 | 0.063 | 0.173 | -0.089 | 0.066 | 0.156 | -0.113 | 0.073 | 0.079 | -0.200 | 0.083 | 0.119 | -0.714 | 0.095 |
| 14 | 0.221 | -0.396 | 0.049 | 0.173 | -0.300 | 0.051 | 0.156 | -0.400 | 0.057 | 0.079 | -0.196 | 0.065 | 0.119 | -0.727 | 0.076 |
| 15 | 0.221 | -0.342 | 0.049 | 0.173 | -0.132 | 0.051 | 0.156 | -0.138 | 0.056 | 0.079 | -0.267 | 0.063 | 0.119 | -0.473 | 0.072 |

Table S3. Author-level coefficients and standard error of Citation Impact by Repeat Authorship for Economics Journals.
