# Supplementary material for: Cumulative advantage and citation performance of repeat authors in scholarly journals
Source: PLoS One. 2022 Apr 13;17(4):e0265831. doi: 10.1371/journal.pone.0265831 (PMC9007338; doi:10.1371/journal.pone.0265831)
Supplement: S4 Table — (DOCX) [file pone.0265831.s004.docx]

| **Publication order** | **Intercept** | **Coefficient** | **Std. Error** | **Intercept** | **Coefficient** | **Std. Error** | **Intercept** | **Coefficient** | **Std. Error** |
| --- | --- | --- | --- | --- | --- | --- | --- | --- | --- |
|  | ***NATURE*** | | | ***PNAS*** | | | ***SCIENCE*** | | |
| 2 | 0.138 | -0.098 | 0.015 | 0.065 | -0.060 | 0.009 | 0.139 | -0.137 | 0.014 |
| 3 | 0.138 | -0.086 | 0.012 | 0.065 | -0.087 | 0.007 | 0.139 | -0.108 | 0.012 |
| 4 | 0.138 | -0.106 | 0.015 | 0.065 | -0.130 | 0.008 | 0.139 | -0.101 | 0.015 |
| 5 | 0.138 | -0.116 | 0.017 | 0.065 | -0.106 | 0.010 | 0.139 | -0.155 | 0.016 |
| 6 | 0.138 | -0.120 | 0.020 | 0.065 | -0.158 | 0.011 | 0.139 | -0.152 | 0.019 |
| 7 | 0.138 | -0.129 | 0.020 | 0.065 | -0.168 | 0.010 | 0.139 | -0.128 | 0.019 |
| 8 | 0.138 | -0.174 | 0.023 | 0.065 | -0.182 | 0.012 | 0.139 | -0.134 | 0.023 |
| 9 | 0.138 | -0.065 | 0.026 | 0.065 | -0.195 | 0.014 | 0.139 | -0.124 | 0.025 |
| 10 | 0.138 | -0.210 | 0.032 | 0.065 | -0.205 | 0.017 | 0.139 | -0.117 | 0.031 |
| 11 | 0.138 | -0.231 | 0.029 | 0.065 | -0.179 | 0.016 | 0.139 | -0.273 | 0.027 |
| 12 | 0.138 | -0.205 | 0.029 | 0.065 | -0.244 | 0.016 | 0.139 | -0.222 | 0.028 |
| 13 | 0.138 | -0.240 | 0.028 | 0.065 | -0.213 | 0.015 | 0.139 | -0.206 | 0.027 |
| 14 | 0.138 | -0.171 | 0.030 | 0.065 | -0.252 | 0.016 | 0.139 | -0.212 | 0.029 |
| 15 | 0.138 | -0.134 | 0.025 | 0.065 | -0.216 | 0.013 | 0.139 | -0.126 | 0.024 |

Table S4. Author-level coefficients and standard error of Citation Impact by Repeat Authorship for *Nature*/*Science*/*PNAS*.
