# Supplementary material for: Cumulative advantage and citation performance of repeat authors in scholarly journals
Source: PLoS One. 2022 Apr 13;17(4):e0265831. doi: 10.1371/journal.pone.0265831 (PMC9007338; doi:10.1371/journal.pone.0265831)
Supplement: S5 Table — (DOCX) [file pone.0265831.s005.docx]

| **Publication order** | **Mean** | **Std. Error** | **Mean** | **Std. Error** | **Mean** | **Std. Error** | **Mean** | **Std. Error** | **Mean** | **Std. Error** |
| --- | --- | --- | --- | --- | --- | --- | --- | --- | --- | --- |
|  | **0-50%** | | **50-75%** | | **75-90%** | | **90-100%** | | **Elite** | |
| 1 | 0.000 | 0.006 | -0.012 | 0.007 | 0.009 | 0.007 | -0.013 | 0.009 | -0.025 | 0.013 |
| 2 | 0.028 | 0.013 | 0.106 | 0.014 | 0.113 | 0.015 | 0.094 | 0.017 | 0.094 | 0.023 |
| 3 | 0.053 | 0.020 | 0.155 | 0.020 | 0.168 | 0.022 | 0.123 | 0.024 | 0.163 | 0.031 |
| 4 | 0.099 | 0.027 | 0.139 | 0.026 | 0.191 | 0.030 | 0.150 | 0.031 | 0.167 | 0.041 |
| 5 | 0.106 | 0.033 | 0.161 | 0.032 | 0.187 | 0.037 | 0.192 | 0.040 | 0.233 | 0.049 |
| 6 | 0.132 | 0.040 | 0.165 | 0.039 | 0.149 | 0.045 | 0.208 | 0.050 | 0.284 | 0.059 |
| 7 | 0.083 | 0.047 | 0.252 | 0.047 | 0.239 | 0.055 | 0.262 | 0.060 | 0.141 | 0.070 |
| 8 | 0.015 | 0.055 | 0.171 | 0.055 | 0.211 | 0.064 | 0.199 | 0.070 | 0.146 | 0.081 |
| 9 | 0.015 | 0.062 | 0.125 | 0.061 | 0.342 | 0.074 | 0.161 | 0.081 | 0.252 | 0.093 |
| 10 | 0.073 | 0.068 | 0.204 | 0.070 | 0.198 | 0.083 | 0.227 | 0.093 | 0.197 | 0.108 |
| 11 | 0.098 | 0.075 | 0.084 | 0.081 | 0.259 | 0.093 | 0.272 | 0.103 | 0.205 | 0.123 |
| 12 | -0.044 | 0.083 | 0.285 | 0.091 | 0.261 | 0.105 | 0.371 | 0.120 | -0.061 | 0.142 |
| 13 | -0.129 | 0.094 | 0.255 | 0.103 | 0.301 | 0.117 | 0.181 | 0.141 | -0.019 | 0.168 |
| 14 | -0.188 | 0.106 | 0.122 | 0.114 | 0.212 | 0.130 | 0.117 | 0.162 | -0.079 | 0.185 |
| 15 | -0.085 | 0.117 | 0.463 | 0.126 | 0.304 | 0.147 | 0.284 | 0.192 | -0.064 | 0.203 |

Table S5. Average Citation Impact by Publication Order for Economics Journals.
