# Supplementary material for: Cumulative advantage and citation performance of repeat authors in scholarly journals
Source: PLoS One. 2022 Apr 13;17(4):e0265831. doi: 10.1371/journal.pone.0265831 (PMC9007338; doi:10.1371/journal.pone.0265831)
Supplement: S6 Table — (DOCX) [file pone.0265831.s006.docx]

| **Publication order** | **Mean** | **Std. Error** | **Mean** | **Std. Error** | **Mean** | **Std. Error** |
| --- | --- | --- | --- | --- | --- | --- |
|  | ***NATURE*** | | ***PNAS*** | | ***SCIENCE*** | |
| **1** | -0.011 | 0.010 | -0.051 | 0.007 | -0.026 | 0.009 |
| 2 | 0.088 | 0.017 | 0.038 | 0.010 | 0.046 | 0.016 |
| 3 | 0.139 | 0.023 | 0.046 | 0.013 | 0.134 | 0.021 |
| 4 | 0.157 | 0.028 | 0.031 | 0.015 | 0.186 | 0.027 |
| 5 | 0.185 | 0.033 | 0.087 | 0.017 | 0.165 | 0.032 |
| 6 | 0.213 | 0.038 | 0.054 | 0.019 | 0.187 | 0.037 |
| 7 | 0.226 | 0.044 | 0.057 | 0.021 | 0.237 | 0.043 |
| 8 | 0.187 | 0.049 | 0.051 | 0.023 | 0.256 | 0.048 |
| 9 | 0.340 | 0.056 | 0.046 | 0.024 | 0.247 | 0.054 |
| 10 | 0.200 | 0.062 | 0.050 | 0.026 | 0.289 | 0.061 |
| 11 | 0.243 | 0.069 | 0.092 | 0.028 | 0.118 | 0.067 |
| 12 | 0.327 | 0.077 | 0.034 | 0.030 | 0.188 | 0.073 |
| 13 | 0.289 | 0.083 | 0.073 | 0.032 | 0.209 | 0.081 |
| 14 | 0.394 | 0.091 | 0.040 | 0.033 | 0.211 | 0.089 |
| 15 | 0.451 | 0.095 | 0.078 | 0.035 | 0.334 | 0.098 |

Table S6. Average Citation Impact by Publication Order for *Nature*/*Science*/*PNAS*.
