# Supplementary material for: Cumulative advantage and citation performance of repeat authors in scholarly journals
Source: PLoS One. 2022 Apr 13;17(4):e0265831. doi: 10.1371/journal.pone.0265831 (PMC9007338; doi:10.1371/journal.pone.0265831)
Supplement: S7 Table — (DOCX) [file pone.0265831.s007.docx]

| **Citation quartile** | **Intercept** | **Coefficient** | **Std. Error** | **Intercept** | **Coefficient** | **Std. Error** | **Intercept** | **Coefficient** | **Std. Error** | **Intercept** | **Coefficient** | **Std. Error** | **Intercept** | **Coefficient** | **Std. Error** |
| --- | --- | --- | --- | --- | --- | --- | --- | --- | --- | --- | --- | --- | --- | --- | --- |
|  | **0-50%** | | | **50-75%** | | | **75-90%** | | | **90-100%** | | | **Elite** | | |
| 2 | -2.468 | 0.300 | 0.008 | -2.157 | 0.283 | 0.010 | -2.143 | 0.290 | 0.012 | -1.806 | 0.246 | 0.015 | -2.106 | 0.301 | 0.027 |
| 3 | -2.468 | 0.464 | 0.015 | -2.157 | 0.462 | 0.017 | -2.143 | 0.483 | 0.020 | -1.806 | 0.411 | 0.023 | -2.106 | 0.518 | 0.041 |
| 4 | -2.468 | 0.536 | 0.033 | -2.157 | 0.653 | 0.035 | -2.143 | 0.727 | 0.038 | -1.806 | 0.627 | 0.043 | -2.106 | 0.856 | 0.059 |

Table S7. Effects of Previous Citation Performance on Likelihood of Future Repeat Authorship for Economics Journals.
