# Supplementary material for: Cumulative advantage and citation performance of repeat authors in scholarly journals
Source: PLoS One. 2022 Apr 13;17(4):e0265831. doi: 10.1371/journal.pone.0265831 (PMC9007338; doi:10.1371/journal.pone.0265831)
Supplement: S8 Table — (DOCX) [file pone.0265831.s008.docx]

| **Citation quartile** | **Intercept** | **Coefficient** | **Std. Error** | **Intercept** | **Coefficient** | **Std. Error** | **Intercept** | **Coefficient** | **Std. Error** |
| --- | --- | --- | --- | --- | --- | --- | --- | --- | --- |
|  | ***NATURE*** | | | ***PNAS*** | | | ***SCIENCE*** | | |
| 2 | -1.381 | 0.309 | 0.020 | -0.485 | 0.204 | 0.012 | -1.518 | 0.326 | 0.019 |
| 3 | -1.381 | 0.466 | 0.041 | -0.485 | 0.379 | 0.024 | -1.518 | 0.482 | 0.038 |
| 4 | -1.381 | 0.591 | 0.048 | -0.485 | 0.453 | 0.028 | -1.518 | 0.616 | 0.044 |

Table S8. Effects of Previous Citation Performance on Likelihood of Future Repeat Authorship for *Nature*/*Science*/*PNAS*.
