# Supplementary material for: Cumulative advantage and citation performance of repeat authors in scholarly journals
Source: PLoS One. 2022 Apr 13;17(4):e0265831. doi: 10.1371/journal.pone.0265831 (PMC9007338; doi:10.1371/journal.pone.0265831)
Supplement: S9 Table — (DOCX) [file pone.0265831.s009.docx]

| **Has chaperone** | **Coefficient** | **Std. Error** | **Coefficient** | **Std. Error** | **Coefficient** | **Std. Error** | **Coefficient** | **Std. Error** | **Coefficient** | **Std. Error** |
| --- | --- | --- | --- | --- | --- | --- | --- | --- | --- | --- |
|  | **0-50%** | | **50-75%** | | **75-90%** | | **90-100%** | | **Elite** | |
| FALSE | -0.023 | 0.010 | -0.072 | 0.012 | -0.082 | 0.014 | -0.130 | 0.017 | -0.093 | 0.028 |
| TRUE | 0.042 | 0.015 | 0.064 | 0.015 | 0.072 | 0.016 | 0.097 | 0.017 | 0.059 | 0.023 |

Table S9. Effect of ‘Chaperone’ Status on Citation Performance for Economics Journals.
