# Supplementary material for: Cumulative advantage and citation performance of repeat authors in scholarly journals
Source: PLoS One. 2022 Apr 13;17(4):e0265831. doi: 10.1371/journal.pone.0265831 (PMC9007338; doi:10.1371/journal.pone.0265831)
Supplement: S10 Table — (DOCX) [file pone.0265831.s010.docx]

| **Has chaperone** | **Coefficient** | **Std. Error** | **Coefficient** | **Std. Error** | **Coefficient** | **Std. Error** |
| --- | --- | --- | --- | --- | --- | --- |
|  | ***NATURE*** | | ***PNAS*** | | ***SCIENCE*** | |
| FALSE | -0.125 | 0.014 | -0.247 | 0.011 | -0.138 | 0.012 |
| TRUE | 0.179 | 0.020 | 0.284 | 0.014 | 0.205 | 0.017 |

Table S10. Effect of ‘Chaperone’ Status on Citation Performance for *Nature*/*Science*/*PNAS*.
