# Supplementary material for: Cumulative advantage and citation performance of repeat authors in scholarly journals
Source: PLoS One. 2022 Apr 13;17(4):e0265831. doi: 10.1371/journal.pone.0265831 (PMC9007338; doi:10.1371/journal.pone.0265831)
Supplement: S1 Fig — Correlation between citation score of consecutive publications for economics journals (left) and Nature/Science/PNAS (right). Each plot includes every pair of consecutive publications in the same journal for the same senior author. (DOCX) [file pone.0265831.s011.docx]

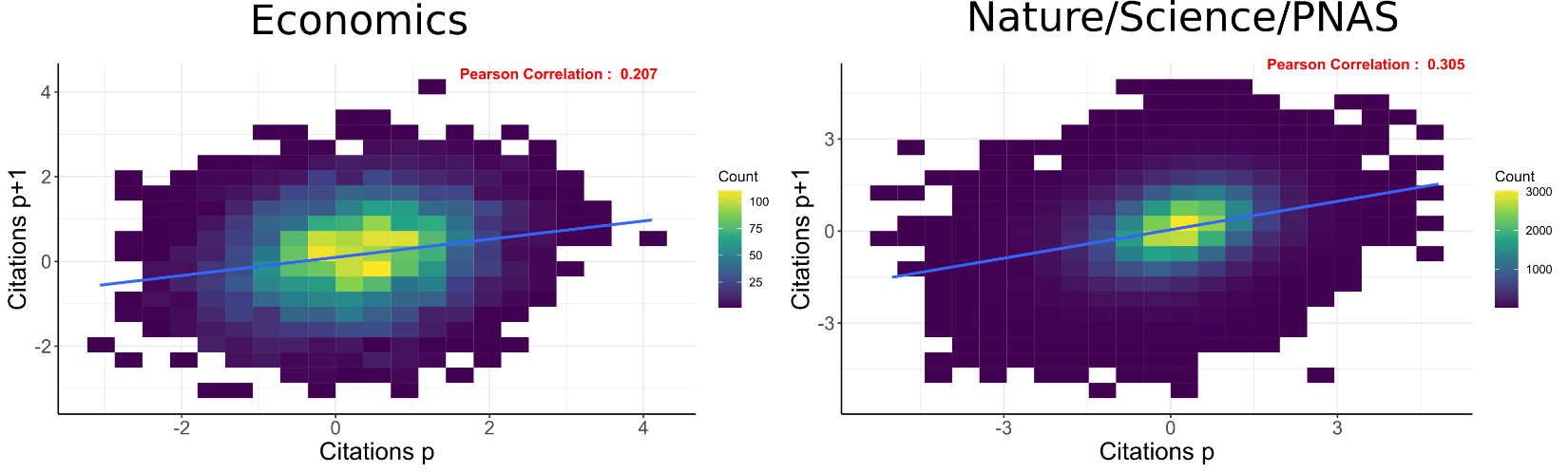


Figure S1. Correlation between citation score of consecutive publications for Economics journals (left) and NSP (right). Each plot includes every pair of consecutive publications in the same journal for the same senior author.
