# Supplementary material for: Cumulative advantage and citation performance of repeat authors in scholarly journals
Source: PLoS One. 2022 Apr 13;17(4):e0265831. doi: 10.1371/journal.pone.0265831 (PMC9007338; doi:10.1371/journal.pone.0265831)
Supplement: Attachment — Submitted filename: PONE_ResRev_Feb1222.docx [file pone.0265831.s012.docx]

RESPONSE TO REVIEWERS:

1) you should justify more convincingly the choice of juxtaposing three multidisciplinary journals with the whole set of economics journals. In my opinion, it should be more ‘natural’ to present a comparison of two research fields rather that a comparison among a field and three multidisciplinary journals. Please note that in economics it is usual to consider some journals, such as the top5, as ‘generalist’, and it is therefore a bit puzzling to have this kind of general comparison. I think that readers would benefit of a better discussion of your choice.

**As per point #1 in Dr. Baccini's last decision letter, we added explanations of the contrast between 'generalist' and 'specialist' journals in the manuscript. We believe the breadth of our journals is a unique feature and strength of the manuscript, as it shows how repeat author dynamics occur in very different scholarly contexts.**

2) you should better justify the choice of considering US only authors. I think that you should discuss this as a limitation of your work.

**As per point #2, we also explained why analyses were limited to US authors. Given linguistic, cultural and institutional differences in universities, disciplines and academic journals, we felt that restricting the analysis to the USA (which was by far the most prolific publishing country) was a 'safe' research decision.**

I think that Figure 1-4 should be modified by adopting inside each Figure identical y-scales for the two panels. The use of different scales may confound readers.

**Additionally, we changed the figures to ensure the axes are on identical scales.**
